# Superior autonomic regulation with ropivacaine over lidocaine in stellate ganglion block for chronic insomnia: a randomized controlled trial

**DOI:** 10.1186/s12871-026-03763-6

**Published:** 2026-03-17

**Authors:** Yongjia Wang, Shuangrui Wang, Xiaoyu Zhang, Xia Li, Xiaomin Fan, Shunqing Hu, Xiangyu Liu, Chaojin Chen, Xinjian Zhang

**Affiliations:** 1 The Third Clinical Medical College, Guangzhou University of Chinese Medicine, Guangdong 510405 Guangzhou, China; 2 Department of Anesthesiology, The Third Affiliated Hospital of Guangzhou University of Chinese Medicine, Guangdong 510378 Guangzhou, China; 3 Department of Anesthesiology, The Third Affiliated Hospital of Sun Yat-sen University, Guangdong 510630 Guangzhou, China

**Keywords:** Stellate ganglion block, Lidocaine, Ropivacaine, Insomnia

## Abstract

**Objective:**

This study aimed to compare the efficacy of lidocaine versus ropivacaine in stellate ganglion block (SGB) for insomnia treatment, including sleep quality improvement, symptom relief, and autonomic nervous system regulation.

**Methods:**

Sixty patients aged 18–75 years with insomnia were randomly divided into the ropivacaine group (Rop group) and lidocaine group (Lido group). Patients in Lido group received a 10-day SGB treatment using 0.8% lidocaine/3 ml. Patients in Rop group were treated with 0.2% ropivacaine/3 ml for SGB for 10 consecutive days. All patients received standard cognitive behavioral therapy. The results of Pittsburgh sleep quality index (PSQI), patient health Questionnaire-9 (PHQ-9), generalized anxiety Disord-7 (GAD-7) and heart rate variability (HRV) were observed and recorded before treatment (T1), the day after treatment completion (T2) and one month after treatment (T3).

**Results:**

Within-group analyses revealed that both groups showed significant improvements in the global PSQI score at T2 and T3, compared to T1 (both *P* < 0.05). Scores for PHQ-9 and GAD-7 also decreased significantly at T2 compared to T1 in both groups (*P* < 0.05). In terms of HRV, the Lido group exhibited significant within-group decreases in RMSSD and SDNN at T2 (*P* < 0.05), while the Rop group showed significant within-group decreases in the LF/HF ratio at T2 (*P* < 0.05). Crucially, between-group comparisons at T2 found no significant differences in PSQI, PHQ-9, or GAD-7 scores (*P* > 0.05). However, a significant between-group difference was observed in the LF/HF ratio, with the Rop group demonstrating a significantly lower value [0.7(0.5,1.2)] compared to the Lido group [1.2(0.9,2.9)] (*P* < 0.05).

**Conclusion:**

SGB treatment with 0.2% ropivacaine and 0.8% lidocaine are equally effective for improving sleep and mood in insomnia. However, ropivacaine’s superior autonomic regulation makes it the preferred choice when mitigating sympathetic hyperactivity is a key therapeutic goal.

**Trial registration:**

This study was registered in the Chinese Clinical Trial Registry (ChiCTR2500098835) on 2025/03/14.

## Background

Chronic insomnia represents a growing public health challenge in contemporary society, with persistent sleep disturbances exerting profound effects on both physical and mental health. Beyond causing daytime fatigue and impaired cognitive function, chronic insomnia significantly increases the risk of developing various comorbid conditions, including anxiety disorders, depression, and cardiovascular diseases [1]. The pathophysiology of insomnia is multifaceted, with dysregulation of the autonomic nervous system—characterized by sympathetic hyperactivity and diminished vagal tone—recognized as a core mechanism [2] . This hyperarousal state disrupts sleep-wake homeostasis, contributing to difficulties with sleep initiation and maintenance.

The management of insomnia currently involves both pharmacological and non-pharmacological therapeutic strategies. Although pharmacotherapy continues to be widely employed, its clinical application is frequently limited by suboptimal efficacy, adverse effects, and potential dependence concerns [3, 4]. Cognitive behavioral therapy for insomnia (CBT-I) is recommended as first-line treatment but faces challenges in accessibility and patient adherence [5–9]. Consequently, there is a compelling need to explore safe and effective alternative interventions.

Stellate ganglion block (SGB) has re-emerged as a promising neuromodulatory technique for treating insomnia [10]. By blocking the cervical sympathetic chain, SGB modulates the hypothalamic-pituitary-adrenal axis and influences the neuro-endocrine-immune network, ultimately promoting restoration of autonomic balance [11–14].

Growing evidence supports the efficacy of SGB in improving sleep quality, particularly in conditions such as post-traumatic stress disorder-related insomnia and perioperative sleep disturbances [10, 15–17]. Both lidocaine and ropivacaine, as amide local anesthetics with favorable cardiac safety profiles, are commonly employed for SGB. However, these agents possess distinct pharmacological characteristics: lidocaine offers rapid onset with intermediate duration of action, while ropivacaine provides longer-lasting sympathetic blockade [17–20]. These pharmacokinetic differences may potentially translate into varying effects on sleep architecture through differential modulation of sympathetic tone, yet no studies have systematically compared their therapeutic efficacy specifically for insomnia.

This knowledge gap has important clinical implications for anesthetic selection in SGB protocols. The choice between these agents is often based on institutional preference rather than evidence specific to sleep disorders, underscoring the need for a direct comparison to inform more precise, personalized treatment approaches. To conduct such a comparison with clinical relevance and validity, it was first essential to establish a safe and physiologically sound dosing regimen. The 3mL of 0.2% ropivacaine was selected due to its superior safety profile [20, 21], demonstrating a lower incidence of hoarseness (6.9%) compared to 4mL (10.3%) in head and neck conditions [22], while avoiding the hemodynamic instability associated with volumes exceeding 5mL [23, 24]. For lidocaine, the 0.8% concentration aligns with current ultrasound-guided clinical practice [25], and was chosen for its capacity to exert systemic regulatory effects beyond local analgesia, including reduction of plasma adrenaline levels [26]. Together, these considerations establish both anesthetic regimens as clinically relevant and physiologically justified for this comparative investigation.

This study utilizes these optimized regimens to systematically compare the efficacy of lidocaine and ropivacaine in SGB for treating insomnia. Our primary focus is to delineate their differential effects on improving sleep quality, alleviating mood symptoms, and modulating the autonomic nervous system. Through this investigation, we aim to establish an evidence-based foundation to guide anesthetic selection in SGB protocols for insomnia management.

## Methods

This study was conducted and reported in accordance with the Consolidated Standards of Reporting Trials (CONSORT) 2010 guidelines.

### Participants

Participant recruitment commenced on March 15, 2025, and concluded on June 15, 2025. Inclusion criteria of the study were: (1) age 18–75 years old, both sexes; (2) meet the diagnostic criteria of chronic insomnia in the International Classification of Sleep Disorders (ICSD-3) [27]; (3) Pittsburgh Sleep Quality Index (PSQI) score ≥ 7; (4) Informed consent was obtained from all patients; Exclusion criteria of the study were: (1) patients allergic to drugs (lidocaine, ropivacaine); (2) patients with disturbance of consciousness or cognitive dysfunction; (3) severe heart block or cardiac insufficiency; (4) abnormal coagulation function or puncture site infection; (5) complicated with other serious organic diseases. Regarding prior medication use, patients who were taking hypnotics, antidepressants, or anxiolytics prior to enrollment continued their original medication regimens throughout the study period.

### Randomisation and interventions

Eligible participants were randomly assigned in a 1:1 ratio to the ropivacaine group (Rop group) and lidocaine group (Lido group). The random allocation sequence was generated by an independent biostatistician using a computer-generated random number list with block sizes of 4 and 6. Allocation concealment was ensured using sequentially numbered, opaque, sealed envelopes, which were prepared and stored by a research assistant not involved in the trial. The enrolling investigator screened participants, obtained informed consent, and then opened the next consecutive envelope to reveal the treatment assignment.

Patients were placed in a supine position with slight neck extension. A high-frequency linear ultrasound probe was placed transversely at the level of the cricoid cartilage. After identifying the C6/C7 transverse processes, longus colli muscle, carotid artery, and vertebral artery, the needle was advanced using an in-plane technique to the fascial plane superficial to the longus colli muscle, lateral to the carotid artery. Following negative aspiration for blood or air, the assigned local anesthetic was injected slowly under direct ultrasound visualization. The Rop group received 3 mL of 0.2% ropivacaine hydrochloride [22–25], while the Lido group received 3 mL of 0.8% lidocaine hydrochloride [21, 26]. The procedure was performed once daily for 10 consecutive days, alternating sides each day. Successful blockade was confirmed by the development of Horner’s syndrome (ptosis, miosis, facial anhidrosis) accompanied by ipsilateral facial warmth and nasal congestion.

All participants received standardized CBT-I as a background intervention. This combined approach of SGB and CBT-I holds the potential to reduce the pharmacological burden on patients [28]. The CBT-I was delivered by a trained psychologist in 10 individual sessions, each lasting approximately 50 min. To ensure consistency and participant convenience, these sessions were conducted on the same days as the SGB procedures, immediately following the block. The intervention followed a standardized manual and included core components such as psychoeducation, stimulus control, sleep restriction, cognitive restructuring, and relaxation training [9, 29].

### Outcomes

The Pittsburgh Sleep Quality Index (PSQI) [30], the Patient Health Questionnaire 9-item Scale (PHQ-9) [31], and the Generalized Anxiety Disorder 7-item Scale (GAD-7) [32] were used to assess sleep quality, anxiety symptoms, and depressive symptoms, respectively. These are previously published and validated questionnaires.

The primary outcome was Pittsburgh sleep quality index (PSQI) [30], which was used as a tool to evaluate the subjective sleep quality of patients. The PSQI scores of the two groups before the 10-day treatment (T1), the day after the 10-day treatment completion (T2), and 1 month after treatment (T3) were recorded. PSQI consisting of 18 participation grade items, including seven aspects, respectively, sleep quality, sleep latency, sleep duration, sleep efficiency, sleep disorder, use of hypnotic drugs and daytime dysfunction, each press 0 ~ 3 points scoring, cumulative each project is the total score of PSQI score, the higher the score worse on behalf of the patient’s quality of sleep.

The secondary outcomes were Patient health Questionnaire-9 (PHQ-9) [31], generalized anxiety disorder scale (GAD-7) [32] and heart rate variability (HRV) [33]. They were used to evaluate the symptoms and severity of depression and anxiety and the function of autonomic nervous system. Records of two groups of patients with T1, T2 time PHQ − 9 and GAD − 7 scores, and the parameters of HRV. HRV parameters included: time domain parameters (SDNN, standard deviation of normal RR intervals in 24 consecutive hours, which mainly reflected total sympathetic and vagal activity; RMSSD, standard deviation of adjacent normal RR intervals, which mainly reflected vagal tone); Frequency domain parameter (LF, low-frequency power, reflecting the common role of the sympathetic and vagus nerve; HF, high frequency power, mainly reflects the vagus nerve tension and LF/HF mainly reflects the sympathetic nerve and vagus nerve of dynamic balance).

All adverse events (AEs) were systematically recorded, including hoarseness, dysphagia, dizziness, and pain at the injection site. Their severity (mild, moderate, or severe) and potential relationship to the intervention were also assessed.

### Blinding

Participants were successfully blinded through the use of identical-appearing local anesthetic solutions and standardized injection procedures. The SGB procedures were performed by a single experienced anesthesiologist who was not involved in the outcome assessments to minimize performance bias. And CBT-I was administered by a qualified physician who had undergone systematic training. To minimize assessment bias, research staff responsible for collecting patient-reported outcome questionnaires (PSQI, PHQ-9, GAD-7) were blinded to group allocation. The statistician performing the final data analysis also remained blinded until completion of the statistical analysis.

### Sample size

Based on a preliminary experiment, sample size calculation was performed using PASS software. With a significance level (α) of 0.05 and statistical power (1-β) of 0.90, the mean PSQI difference between groups was estimated to be 2.49. The standard deviations were 2.59 for Rop group and 3.16 for Lido group. Assuming a 1:1 allocation ratio, the calculation yielded a requirement of 24 participants per group. To account for an anticipated 20% dropout rate, a minimum of 30 participants per group (60 total) were required.

### Statistical methods

Statistical analysis was conducted using SPSS Statistics 26.0 software. Count data were expressed as the number of cases or percentages, and comparisons between groups were analyzed using the χ2 test. Measurement data that followed a normal distribution were expressed as (x ± s), and comparisons within and between groups were conducted using t-tests. For measurement data that did not follow a normal distribution, the median (interquartile range) was used for presentation, and comparisons between groups were performed using the Mann-Whitney U test, while comparisons within groups were conducted using the Wilcoxon rank sum test. A difference was considered statistically significant when *P* < 0.05.

## Results

### Participant flow and recruitment

**Fig. 1:**
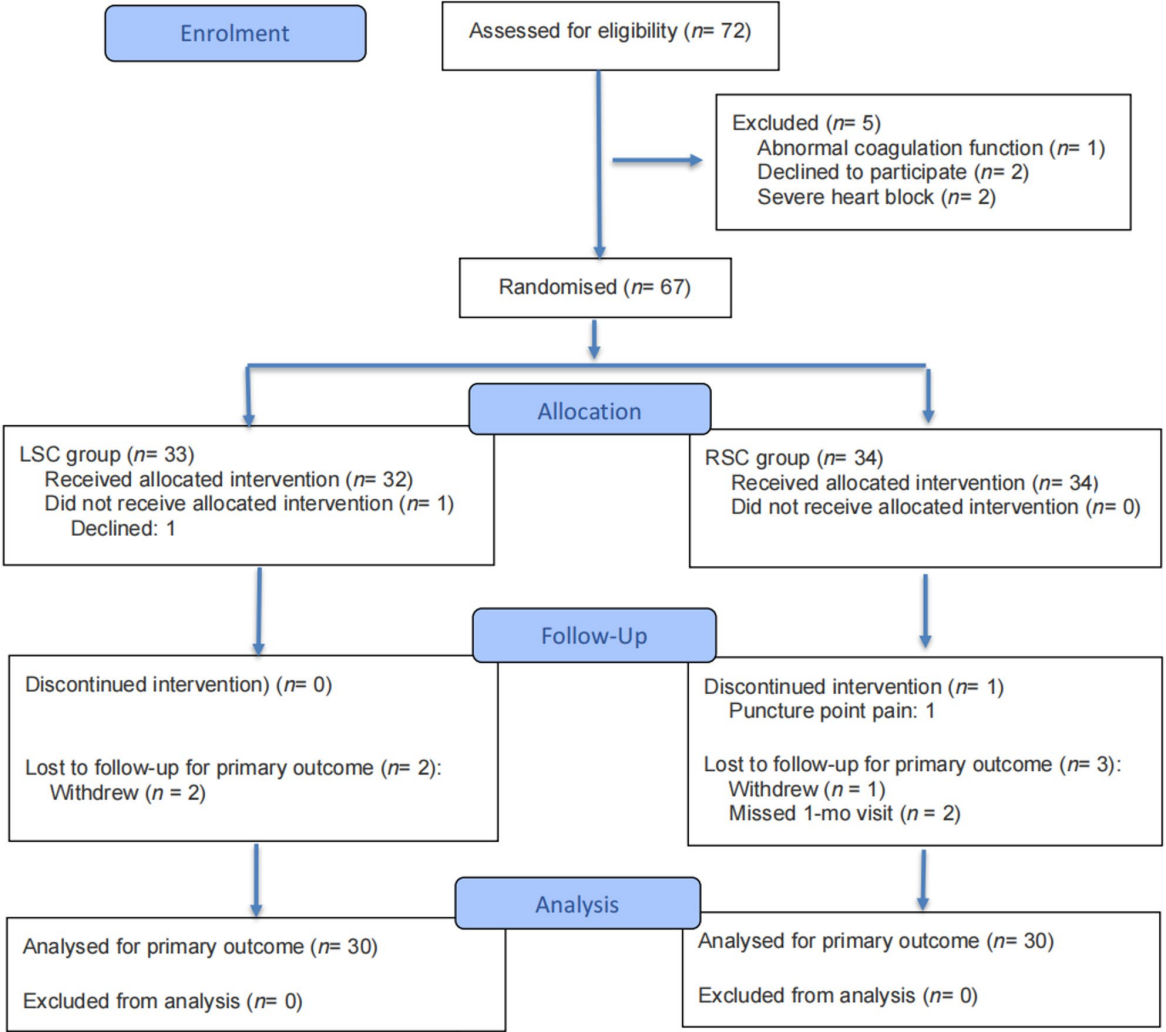
CONSORT flowchart of patient disposition in the study

Figure 1 shows the flow of participants through the trial. A total of 67 patients were initially accessed in this study, among whom 4 patients withdrew voluntarily and 3 patients provided invalid data for the scale, with a dropout rate of 11.67%. Finally, 60 patients were enrolled, with 30 patients in each group.

### Baseline data

There was no significant difference in age, gender, height, BMI and insomnia duration between the two groups (*P* > 0.05), as shown in Table 1.

**Table 1:** Baseline characteristics of participants

General information	Lido group (*n* = 30)	Rop group (*n* = 30)	t/χ^2^ values	*P*-value
Age/years	48.80 ± 12.45	52.30 ± 10.43	-1.170	0.247
Gender /[*n* (%)]				
male	15 (50)	11 (36.7)	1.086	0.297
female	15 (50)	19 (63.3)		
Height /cm	167.13 ± 7.7	164.73 ± 6.0	1.361	0.179
BMI/ (kg/m2)	22.014 ± 2.9	22.556 ± 2.3	-0.716	0.477
Duration of insomnia/year	8.80 ± 8.36	9.33 ± 7.55	-0.259	0.796

Data are presented as mean ± standard deviation or number (percentage)

*BMI* body mass index

Between-group comparisons were performed using independent t-tests for continuous variables and the Chi-square test for gender. *P*-value > 0.05 indicates no significant difference between groups at baseline

### Primary outcome: PSQI scores

At baseline (T1), no statistically significant differences were observed in the global PSQI score or any of its component scores between the two groups (*P* > 0.05, Tables 2 and 3).

**Table 2:** Comparison of Pittsburgh Sleep Quality Index (PSQI) total scores

Groups	T1	T2	T3
Lido Group	17.23 ± 2.459	13.13 ± 3.082^*^	14.87 ± 3.126^*^
Rop Group	16.67 ± 2.294	13.40 ± 3.529^*^	15.10 ± 2.734^*^

Data are presented as mean ± standard deviation

*P < 0.05 compared with T1 within the same group (paired t-test). No significant differences were found between groups at any time point (independent t-test, *P* > 0.05)

**Table 3:** Comparison of PSQI dimension scores

Indicators	Groups	T1	T2	T3
Quality of sleep	Lido group	2.73 ± 0.450	1.83 ± 0.699^*^	2.20 ± 0.714^*^
	Rop group	2.80 ± 0.407	2.00 ± 0.830^*^	2.33 ± 0.606^*^
Sleep latency	Lido group	2.67 ± 0.711	2.33 ± 0.922^*^	2.57 ± 0.679
	Rop group	2.90 ± 0.481	2.27 ± 0.785^*^	2.47 ± 0.730^*^
Sleep duration	Lido group	2.73 ± 0.521	2.17 ± 0.699^*^	2.33 ± 0.661^*^
	Rop group	2.63 ± 0.669	2.27 ± 0.740^*^	2.37 ± 0.718^*^
Sleep efficiency	Lido group	2.73 ± 0.691	2.03 ± 1.033^*^	2.37 ± 0.765^*^
	Rop group	2.63 ± 0.718	2.07 ± 1.172^*^	2.50 ± 0.731
Sleep disturbances	Lido group	1.43 ± 0.679	1.17 ± 0.461^*^	1.27 ± 0.640
	Rop group	1.37 ± 0.615	1.13 ± 0.571^*^	1.20 ± 0.551
Use of hypnotics	Lido group	2.57 ± 1.006	2.03 ± 1.377^*^	2.10 ± 1.322^*^
	Rop group	2.17 ± 1.234	2.07 ± 1.337	2.03 ± 1.326
Daytime dysfunction	Lido group	2.37 ± 0.928	1.53 ± 1.167^*^	2.00 ± 0.910
	Rop group	2.20 ± 1.031	1.60 ± 1.102^*^	2.20 ± 0.847

Data are presented as mean ± standard deviation

*P < 0.05 compared with T1 within the same group (paired t-test). No significant differences were found between groups at any time point for any component (independent t-test, *P* > 0.05)

In the Lido group, scores for sleep quality, sleep duration, sleep efficiency, use of hypnotics, and daytime dysfunction were significantly lower at both T2 and T3 compared to T1 (*P* < 0.05). The sleep latency score was significantly reduced at T2, but not at T3. In the Rop group, scores for sleep quality, sleep latency, sleep duration, and daytime dysfunction were significantly lower at both T2 and T3 compared to T1 (*P* < 0.05). The sleep efficiency score was significantly reduced at T2, but not at T3. Sleep disturbances scores significantly decreased at T2 in both groups compared to T1 (*P* < 0.05), but these reductions were not maintained at T3. The score for use of hypnotics in the Rop group did not show a statistically significant change at either T2 or T3.

Despite the significant within-group improvements, no statistically significant differences were found in the global PSQI score or any of the seven component scores between the Lido and Rop groups at either the T2 or T3 time points (*P* > 0.05).

### Secondary outcomes

#### PHQ-9 and GAD-7 scores

As presented in Tables 4 and 5, the baseline (T1) PHQ-9 and GAD-7 scores were comparable between the Lido and Rop groups (*P* > 0.05).

**Table 4:** Comparison of PHQ-9 scores

Groups	T1	T2
Lido Group	10.37 ± 6.667	6.17 ± 4.997^*^
Rop group	10.63 ± 5.641	8.07 ± 6.186^*^

Data are presented as mean ± standard deviation

*P < 0.05 compared with T1 within the same group (paired t-test). No significant difference was found between groups at T2 (independent t-test, *P* > 0.05)

**Table 5:** Comparison of GAD-7 scores

Groups	T1	T2
Lido Group	7.90 ± 6.166	5.00 ± 4.727^*^
Rop group	9.17 ± 6.254	7.10 ± 6.255^*^

Data are presented as mean ± standard deviation

*P < 0.05 compared with T1 within the same group (paired t-test). No significant difference was found between groups at T2 (independent t-test, *P* > 0.05)

Following the intervention, at the T2 time point, both groups exhibited a statistically significant reduction in both PHQ-9 and GAD-7 scores compared to their respective baseline values (*P* < 0.05). This indicates a significant alleviation of depressive and anxiety symptoms in both groups after treatment. When comparing the two groups directly at T2, no statistically significant difference was found in either the PHQ-9 or GAD-7 scores (*P* > 0.05), suggesting a similar magnitude of improvement in depressive and anxiety symptoms between the lidocaine and ropivacaine groups.

### HRV parameters

At baseline (T1), all measured HRV parameters, including RMSSD, SDNN, and LF/HF, showed no significant differences between the Lido and Rop groups (*P* > 0.05), as detailed in Table 6.

**Table 6:** Comparison of HRV parameters

	Group	T1	T2
RMSSD	Lido group	34.4(21.9,43.2)	30.0(21.9,39.5)^*^
	Rop group	27.0(19.5,32.2)	23.2(20.4,30.9)
SDNN	Lido group	28.0(19.3,37.7)	22.2(17.5,30.1)^*^
	Rop group	21.3(15.3,29.6)	19.4(14.1,28.7)
LF/HF	Lido group	1.7(0.8,2.9)	1.2(0.9,2.9)^†^
	Rop group	1.1(0.5,2.2)	0.7(0.5,1.2)^*^

Data are presented as median (interquartile range)

*RMSSD* root mean square of successive RR interval differences, *SDNN *standard deviation of all normal RR intervals, *LF *low-frequency power, *HF *high-frequency power

*P < 0.05 compared with T1 within the same group (Wilcoxon signed-rank test)

^†^*P* < 0.05 for between-group comparison at T2 (Mann-Whitney U test)

After treatment (T2), distinct patterns of change were observed within each group: In the Lido group, significant decreases from T1 to T2 were observed in RMSSD and SDNN values (*P* < 0.05). In the Rop group, significant decreases from T1 to T2 were observed in LF/HF value (*P* < 0.05). Crucially, the between-group comparison at T2 revealed a statistically significant difference in the LF/HF ratio (*P* < 0.05). The LF/HF value in the Rop group was significantly lower than that in the Lido group at the end of treatment.

### Safety and adverse events

The safety profiles of both interventions are summarized in Table 7. During the treatment period, a total of 23 adverse events were documented across 600 SGB procedures (300 per group). All reported events were mild and self-limiting, resolving spontaneously without medical intervention. No severe complications or serious adverse events (SAEs) were observed.

**Table 7:** Adverse events during treatment period

Group	Treatment (times)	Hoarseness (times)	Dysphagia (times)	Dizziness (times)	Puncture point pain (times)
Lido group	300	3	2	2	4
Rop group	300	4	1	1	5

Data are presented as the number of events

*AE *adverse event

No significant differences were found between groups for any AE category or total AEs (Fisher’s exact test, *P* > 0.05)

The adverse events consisted of hoarseness (7 cases), dysphagia (3 cases), dizziness (3 cases), and pain at the puncture site (9 cases). The incidence of each adverse event type, as well as the overall adverse event rate, was comparable between the Lido and Rop groups, with no statistically significant differences (*P* > 0.05).

## Discussion

This prospective randomized controlled trial provides the first direct comparison of lidocaine versus ropivacaine for SGB in the treatment of chronic insomnia. Our findings yield two principal conclusions: first, both local anesthetics demonstrate comparable efficacy in improving subjective sleep quality and alleviating mood symptoms; second, despite this clinical equivalence, the agents exert distinct patterns of autonomic modulation, with ropivacaine showing superior efficacy in reducing sympathetic predominance as measured by the LF/HF ratio.

The absence of significant between-group differences in global PSQI scores, despite the differing pharmacological profiles of lidocaine and ropivacaine, merits careful interpretation. This equivalence likely stems from two interrelated aspects of our study design. First, the robust and synergistic effect of the combined SGB and CBT-I intervention may have induced a state of therapeutic saturation [5, 9, 28], thereby precluding the detection of statistically discernible differences between the two anesthetics. Second, the protocol of repeated daily SGB sessions over 10 consecutive days [19, 20] may have effectively mitigated the pharmacodynamic differences in duration of action. By ensuring sustained sympathetic blockade in both groups, this regimen could have compensated for lidocaine’s shorter half-life, rendering the prolonged effect of a single ropivacaine injection less critical for cumulative sleep outcomes. However, in the absence of direct pharmacokinetic data, this interpretation remains a hypothesis warranting further investigation; it is possible that the cumulative effect of repeated blocks simply overshadowed the differential duration of action of single injections.

Although no significant between-group differences were found in PSQI subscales, the patterns of improvement (e.g., greater reduction in sleep latency with ropivacaine and sleep efficiency with lidocaine) may hint at differential mechanisms of action. Ropivacaine’s longer duration could preferentially improve sleep onset, while lidocaine’s rapid onset might affect sleep maintenance. Future studies should explore these subtleties using objective measures.

The most physiologically significant finding of our study is the differential effect on HRV parameters, particularly the LF/HF ratio. The ropivacaine group achieved a significantly greater reduction in this key index of sympathovagal balance. This can be attributed to ropivacaine’s distinct pharmacological profile: its higher lipid solubility, greater protein binding capacity, and longer duration of action facilitate more profound and sustained blockade of sympathetic ganglionic transmission [33]. While lidocaine produced a broader, non-specific reduction across multiple HRV domains—possibly reflecting a generalized dampening of autonomic tone—ropivacaine induced a more targeted shift toward parasympathetic dominance. This autonomic regulatory superiority of ropivacaine carries important clinical implications beyond sleep improvement. Chronic insomnia is frequently comorbid with conditions characterized by autonomic dysfunction, including anxiety disorders, depression, and cardiovascular diseases [1, 34–38]. Sympathetic hyperactivity, as indicated by an elevated LF/HF ratio, is a recognized risk factor for hypertension, arrhythmias, and adverse cardiovascular events. Therefore, for insomnia patients with documented autonomic imbalance or significant cardiovascular comorbidities, ropivacaine may offer dual benefits: improving sleep while concurrently addressing maladaptive sympathetic overactivity.

Our findings support a more nuanced, phenotype-guided approach to local anesthetic selection for SGB in insomnia. We propose that HRV assessment could serve as a practical biomarker to inform clinical decision-making. For patients with elevated baseline LF/HF ratios or prominent symptoms of sympathetic arousal (e.g., palpitations, restlessness), ropivacaine may be the preferred agent due to its superior autonomic regulatory effects. Conversely, for patients with relatively normal autonomic function or when rapid onset is desired, lidocaine remains an excellent and effective option. Furthermore, considering the pharmacokinetic differences, treatment schedules could be optimized: more frequent sessions might be planned for lidocaine (e.g., 3–4 times weekly) to maintain continuous effect, while ropivacaine’s longer action might permit less frequent administration (e.g., 1–2 times weekly), potentially improving convenience and cost-effectiveness.

The comparable incidence of self-limiting adverse events between groups demonstrates that both ropivacaine and lidocaine exhibited similar safety profiles for SGB in this study. Crucially, this finding indicates that despite their substantial differences in duration of action, the pharmacological disparity did not translate into different safety outcomes when the blocks were performed by an experienced operator using standardized ultrasound-guided technique. Therefore, the difference in anesthetic duration does not impact the safety profile of SGB procedures when performed by skilled practitioners. These findings are consistent with previous studies [20, 39, 40], further confirming the comparable safety of both local anesthetics for SGB.

Several limitations should be considered when interpreting our results. First, the reliance on subjective patient-reported outcomes introduces potential recall and reporting bias; future studies would benefit from incorporating objective measures such as polysomnography or actigraphy. Second, the one-month follow-up limits assessment of long-term treatment durability and autonomic changes. Future studies with extended follow-up (e.g., 6 or 12 months) are warranted to assess the sustainability of SGB effects and guide long-term treatment planning. Third, as a single-center study, our findings require validation in larger, multi-center trials. Additionally, the pharmacological equivalence of the two anesthetic regimens remains a key methodological consideration. Although the concentrations (0.2% ropivacaine vs. 0.8% lidocaine) were selected based on established safety data and clinical usage, they may not represent matched pharmacodynamic profiles in terms of onset, intensity, or duration of sympathetic blockade.

In conclusion, this randomized trial demonstrates that both 0.2% ropivacaine and 0.8% lidocaine are safe and effective for SGB in the treatment of chronic insomnia, producing equivalent improvements in sleep quality and mood symptoms. However, ropivacaine exhibits a distinct advantage in modulating autonomic balance, specifically in reducing sympathetic predominance. These findings suggest that the choice between these local anesthetics should not be based solely on sleep outcomes but should consider the patient’s autonomic profile and therapeutic goals. For individuals where correction of autonomic dysfunction is paramount, ropivacaine appears to be the superior choice. This study advances the field toward more personalized, physiology-informed application of neuromodulatory therapies for sleep disorders.

## Data Availability

The data that support the findings of this study are available from the corresponding author upon reasonable request.

## Abbreviations

AE: Adverse event
BMI: Body mass index
CBT-I: Cognitive behavioral therapy for insomnia
GAD-7: Generalized anxiety disorder-7
HF: High-frequency power
HRV: Heart rate variability
ICSD-3: International classification of sleep disorders, 3rd edition
LF: Low-frequency power
Lido Group: Lidocaine group
PHQ-9: Patient Health Questionnaire-9
PSQI: Pittsburgh Sleep Quality Index
RMSSD: Root mean square of successive RR interval differences
Rop Group: Ropivacaine group
SAE: Serious adverse event
SDNN: Standard deviation of all normal RR intervals
SGB: Stellate ganglion block
T1: Before treatment
T2: The day after treatment completion
T3: One month after treatment
